# Effect and molecular mechanism research of *Astragalus membranaceus* on inhibiting intestinal absorption of six alkaloids of *Aconitum carmichaelii* in spleen deficiency rats

**DOI:** 10.1016/j.chmed.2021.07.001

**Published:** 2021-07-09

**Authors:** Xulong Chen, Xinli Liang, Xiaoqiang Kong, Miaomiao Ji, Abid Naeem, Cheng Li, Hao Zheng, Mingxia Gao, Zhenggen Liao

**Affiliations:** a Key Laboratory of Modern Preparation of Traditional Chinese Medicine, Ministry of Education, Jiangxi University of Traditional Chinese Medicine, Nanchang 330004, China; b Affiliated Hospital, Jiujiang University, Jiujiang 332000, China

**Keywords:** alkaloids, *Aconitum carmichaelii* Debx., *Astragalus membranaceus* (Fisch.) Bge., BCRP, intestinal absorption, MRP2, spleen deficiency, tight junction

## Abstract

**Objective:**

To investigate the effect and the mechanism of *Astragalus membranaceus* (Huangqi in Chinese, HQ) extract on the intestinal absorption of six alkaloids of *Aconitum carmichaelii* (Fuzi in Chinese, FZ) in rats with spleen deficiency and provide novel insights into the application of HQ on modulating intestinal barrier.

**Methods:**

Four-week-old male Sprague-Dawley rats were fed with Xiaochengqi Decoction to induce the spleen deficiency model for 40 d. Single-pass intestinal perfusion model were used to study the effects of HQ extract on the absorption of alkaloids. Protein expression and mRNA levels of MRP2 and BCRP and tight junction proteins (TJ, including Claudin-1, Occludin and ZO-1) were measured using Western blot and real-time PCR, respectively. The location and expression of TJ protein was also investigated by the immunofluorescence method.

**Results:**

Compared with the normal group, the protein expression of MRP2, BCRP and TJ proteins in the model group were significantly down-regulated. After oral administration of HQ, the alkaloid absorption in intestinal villi was inhibited, MRP2, BCRP and TJ proteins were up-regulated, the green fluorescence staining of Claudin-1, Occludin, and ZO-1 was enhanced, and a thick layer of mucus was deposited on the surface of the epithelium of the intestinal cavity.

**Conclusion:**

HQ as an intestinal barrier modulator improves the physiological changes of the intestinal environment of spleen deficiency to reduce the absorption of toxic components, leading to a decrease in the absorption of drug-like molecules.

## 1 Introduction

*Astragalus membranaceus* (Fisch.) Bge. (Huangqi in Chinese, HQ) is often used as a *qi*-invigorating herb in traditional Chinese medicine. It is thought to exert function through invigorating the spleen and lung, improving spleen function with an extensive and a long history clinical application (Chen, Tsai, Lee, Liu, & Tsai, 2014). In traditional Chinese medicine, the spleen is considered as an important immune organ with the functions of nourishing and sustaining life (Wu et al., 2014, Yu et al., 2014). At the same time, it also has the functions of defending the body against diseases, the external environment and maintaining core body processes at equilibrium (De Matos Filho & Petroianu, 2015). It has been reported that medicine with efficacy of replenishing *qi* and invigorating spleen can repair the mucosal barrier structure and balance the intestinal flora of spleen deficiency animals, and reduce the intestinal permeability of toxic substances such as endotoxins (Ma et al., 2015, Zhang et al., 2014a, Zhang et al., 2014b). The results indicate that spleen is closely related to the intestinal barrier, reflecting the theory of “the spleen being as the guard of the human body” in TCM. Modern medicine believes that the small intestine is the first barrier to defend the body against ingested harmful substances (Grant et al., 2015). Since the spleen has a key role in maintaining the body equilibrium, we considered the possibility that the spleen is involved in supporting the small intestine to block absorption of harmful substance.

*Aconitum carmichaelii* Debx. (Fuzi in Chinese, FZ) is known as the first drug to revive the *yang* for resuscitation and has played an important role in disease control since ancient times. FZ treatment leads to effects of expanding blood vessels, reducing inflammation as well as having anti-tumor properties (Wu et al., 2018). Along with the widespread use of FZ, it is regularly used in combination with other traditional Chinese medicines and/or modern drugs (Peter et al., 2013), increasing the chance of adverse drug interactions and changing drug absorption properties in combination (Zhang et al., 2016, Zhu et al., 2014). HQ and FZ are commonly used as a herbal pair, such as in the Qifu Decotion, a famous preparation in traditional Chinese medicine first mentioned in “*Wei's Family Prescription*” (Song Dynasty). However, texts such as “The explanation of medicinal processing of Leigong” (Shi-cai Li, Ming Dynasty) and the book of “*Medica Truth*” (Qing Dynasty) indicate that the efficacy of FZ is reduced in the presence of HQ. Based on these scripts, modern research shows that HQ can reduce the toxicity of FZ (Zhang et al., 2018), but the mechanism by which HQ limits FZ activity is not known. We hypothesized that HQ, through its activation of splenic function, could impact the absorption of FZ and thus reduce its potency.

After oral administration of the drug, the pharmacological effect of the drug depends on amount of absorption. While there are many barriers in absorption process, among them efflux drug transporter and tight junctions are considered the most important absorption barriers. Efflux drug transporter mainly includes P-gp, BCRP and MRP2 (Wu et al., 2018), these three transporter proteins all need energy which is supplied by adenosine triphosphate (ATP) hydrolysis. These proteins are expressed on the intestinal epithelial mucosa lateral and are responsible for effluxing drugs, toxicity substance, and other exogenous substances outside to the lumen. The tight junction of the small intestinal mucosa is made up of occludin, claudin, ZO-1, ZO-2, ZO-3 and cytoskeletal protein (Ali et al., 2014, Arias et al., 2014). The permeability of small intestinal mucosa will affect the amount of drug in the blood, while intestinal mucosa’s permeability may change under certain conditions. In our previous study (Kong et al., 2017), we found that HQ could significantly decrease the intestinal absorption of six alkaloids of FZ in normal rats. Studies also have shown that six alkaloids of FZ were substrate of P-gp, BCRP, MRP2, so their absorption process *in vivo* was regulated by these transporters (Wu et al., 2017, Ye et al., 2013). However, whether HQ can also inhibit the absorption of alkaloids of FZ under spleen deficiency syndrome required further investigation, this study was conducted to evaluate the effects of HQ on the activity of intestinal epithelial cell efflux drug transporter and tight junction formation in rats with spleen deficiency syndrome.

## 2 Materials and methods

### 2.1 Materials and chemicals

*A. membranaceus* (batch: 161213) and *A. carmichaelii* (batch: 20150601) were all purchased from Jiangxi Jiangzhong Traditional Chinese Medicine Decoction Co., Ltd. (Jiangxi, China), and were identified by Professor Shou-wen Zhang of Jiangxi University of Traditional Chinese Medicine. Reference substance of hypaconitine, mesaconitine, aconitine, benzoylhypaconine, benzoylmesaconine, benzoylaconine and astragaloside IV were obtained from National Institutes for Food and Drug Control (Beijing, China).

### 2.2 Main reagents

Phosphate buffer saline (PBS), Coomassie brilliant blue G-250, albumin from bovine serum (BSA) and SDS-PAGE Gel preparation kit were all purchased from Beyotime Biotechnology (Shanghai, China). 5X protein loading buffer and Western transfer buffer were bought from Beyotime Biotechnology (Shanghai, China). RIPA lysis buffer, protease inhibitors, phosphatase inhibitors, the BCA Protein Assay reagent kit, and the ECL kit were purchased from Beijing Com Win Biotech Co., Ltd. (Beijing, China). A rabbit monoclonal anti-MRP2 antibody, a rabbit monoclonal anti-BCRP antibody were all purchased from Abcam (Cambridge, UK). A rabbit monoclonal anti-Claudin-1 antibody, a rabbit monoclonal anti-Occludin antibody and a rabbit monoclonal anti-ZO-1 antibody were all purchased from Boster Biological Technology Co., Ltd (Wuhan, China). The GAPDH mouse monoclonal antibody was purchased from Shanghai Bioleaf Biotech Co., Ltd. (Shanghai, China). Horseradish peroxidase (HRP)-conjugated goat anti-mouse IgG, and HRP-labeled goat anti-rabbit IgG (H + L) was purchased from Beyotime Biotechnology (Shanghai, China). Trizol RNA extraction kit was obtained from Ambion (Thermo Fisher Scientific, Beijing, China), and the reverse transcription kit was purchased from Promega (Madison, WI, USA). Power SYBR Green PCR Mix was procured from Life Technologies, Inc. And PCR primers were synthesized by Shanghai Sangon Biotech Co., Ltd. (Shanghai, China). All other chemicals and solvents were of analytical grade or better.

### 2.3 Establishment of spleen deficiency syndrome model

Male Sprague-Dawley rats (200–220 g, body weight) for the intestinal absorption study were obtained from the animal center of Jiangxi University of Traditional Chinese Medicine (Nanchang, China). The animals were housed in the room which was well ventilated and had a regular 12:12 h light/dark cycle throughout the experimental period, and then were randomly divided into the normal group and model group. Each rat in the model group was fed 5 mL of Xiao Chengqi Decoction (Jiang, Huang, Kong, & Liao, 2017) every day, and the diet was given at 9 AM and removed at 9 PM. The model development took 40 d. All the animal studies were done in accordance to the approved protocols and guidelines of the Institutional Animal Ethical Care Committee (NIPER) (Sharma, Varma, Chawla, & Panchagnula, 2005). All efforts were made to minimize the number of animals used and their suffering. Finally, we evaluated the spleen deficiency model by observing the appearance symptoms and measuring *D*-xylitol and amylase in rats.

Determination of serum *D*-xylose content: After 24 h of fasting, rats in each group were gavaged with 6 g/kg (concentration 0.3 g/mL) *D*-xylose suspension. Then after 1 h of gavage, blood samples were collected from the retro-orbital venous plexus of rats and placed in heparinized centrifuge tubes, and the samples were centrifuged at 4000 rpm for 5 min to retrieve the upper plasma, which was stored in a low-temperature refrigerator in a pip-bottom tube. The test was performed following the instructions provided with the xylose kit. The content of serum *D*-xylose (mmol/mL) = (OD value of the measuring tube − OD value of the fixed blank tube)/(OD value of the standard tube − OD value of the reagent blank tube) × standard concentration (the content unit of *D*-xylose was mmol/L and the standard concentration was 1.33 mmol/L).

Determination of serum amylase activity: After 24 h of fasting, blood samples were collected from the retro-orbital venous plexus of rats and placed into heparinized centrifuge tubes, and the samples were centrifuged at 4000 rpm for 5 min to retrieve the upper plasma and stored in a sharp bottom tube in a low-temperature refrigerator for serum xylose content to be measured. After dilution 128 times, followed the instructions provided with the xylose test box and reagents. AMS activity (U/dL) = (blank OD value − measured OD value)/blank OD value × 80 × dilution before sample test.

### 2.4 Preparation of water extract of *A. membranaceus* and *A. carmichaelii*

HQ and FZ decoction were all prepared according to the previous study (Kong et al., 2017). The contents of the main bioactive compounds in HQ and FZ were also determined via HPLC external standard method and the contents of pureonebio and astragaloside IV in HQ extract were 0.034% and 0.225%, respectively. The contents of monoester alkaloids and diester alkaloids in FZ extract were 4.56% and 0.40%, respectively, and both types of extracts were stored at −20 °C for later use.

### 2.5 Effect of HQ on intestinal absorption of six alkaloids of FZ by single-pass perfusion model in spleen deficiency rats

Fasted (16–18 h) male Sprague-Dawley spleen deficiency rats and normal rats were anesthetized with 10% chloral hydrate (3.4 mL/kg by intraperitoneal injection). Single-pass intestinal perfusion studies were carried out using an experimental design similar to that described by Xin-li Liang (Liang et al., 2012). The rats were fixed on a plate, and body temperature was maintained at 37 °C using an overhead work-light and a heating mat. The abdomem was opened along the middle line of the abdomen and the intestinal segment to be inspected was separated. The intestinal segment was kept moist throughout the experiment by gently applying buffer using cotton wool balls saturated with warm saline and was washed cleanly using constant temperature physiological saline at a flow rate of 5 mL/min. Then, 50 mL of the test solutions were preheated to 37 °C and were irrigated for 10 min at a flow rate of 1 mL/min, and then balanced 30 min at a flow rate of 0.2 mL/min. Furthermore, the perfused samples were collected at 15, 30, 45, 60, 75, 90, 105, 120 min and at the same time perfusate samples were assayed for drug content by LC-MS. Blood samples were taken at 120 min from the hepatic portal vein, centrifuged to separate the plasma, and the plasma was then analyzed using LC–MS analysis. Finally, the rats were euthanized, and the perfused intestinal segments were cut out, and its length and internal diameter were measured.

The characterization of intestinal absorption of six alkaloids of FZ and the intestinal absorption effects of HQ extracts on them were studied by using the above experimental technique. The correction for water flux was done in outgoing drug concentration by the method described by Sutton et al. (Ali et al., 2014). The intestinal permeability (*P*_app_) of drugs were determined by using the following mathematical equation (Dixit, Jain, & Dumbwani, 2012).

where, r is the luminal radius of intestinal segment and l is the mean length of the perfused duodenum segment. C_out(corr)_ is the correct content of the drug solution of exit, C_out_ is the content of the drug solution of exit; C_in_ is the perfusion drug content of the intestinal inlet (μg/mL), and Q is the perfusion rate (mL/min).

### 2.6 Dosage regimen and intestinal tissue sample processing of spleen deficiency rats

Total 20 SD spleen deficiency rats with weights of 180–220 g were randomly divided into four groups, spleen deficiency model group, low dose group (3.33 g HQ crude drug/kg), medium dose group (10 g HQ crude drug/kg) and high dose group (30 g HQ crude drug/kg), respectively. At the same time, five rats with weight of 180–220 g were randomly selected from the normal rats as normal group. Rats were administered continuously for 7 d and were sacrificed on the eighth day, and the duodenum (10 cm) was taken, washed, cleaned, and then was stored at − 80 °C after cutting into segments. Also, rats ileum (10 cm) was taken and washed cleanly by precooled PBS buffer (4 °C), and then was put in embedding box after cutting into segments, and the box was immersed in 4% polyformaldehyde solution and was stored at 4 °C refrigerator.

### 2.7 Effect of HQ extract on expression of MRP2 and BCRP and tight junction (Claudin-1, Occludin, ZO-1)

Small pieces of duodenal tissues was precisely weighed and disintegrated using RIPA lysate for 30 min, and then were centrifuged for 5 min at a speed of 12 000 rpm. The protein content of the supernatant was determined according to the instructions of the BCA kit. Protein samples were mixed with 5 × loading buffer and were denatured at 100 °C for 3 min. Moreover, an equal amount of protein (60 µg) was separated by SDS-PAGE (5% and 10% stacking and separating gels, respectively) and then subsequently transferred from gel to the PVDF membrane. After blocking for 2 h with non-fat milk, the corresponding antibodies with proper dilution were added and incubated with the membrane at 4 °C overnight. The membrane was washed before incubation with the corresponding second antibody at a proper dilution in the same buffer for 1 h at room temperature. Western blot signals were determined by an ECL chemiluminescence detection agent and the relative intensity of each protein band was scanned and half quantified by ImageJ software.

### 2.8 Real-time PCR analysis

Total RNA of small pieces of duodenal tissue was extracted by Trizol kits, and its contents were determined via the optical density (OD) value at the wavelength of 280 nm by the enzyme micro-plate reader. Total RNA was dyed with 1.0% agarose gel electrophoresis and 0.5 g/mL ethidium bromide and was observed at three bands of 28S, 18S, 5S under ultraviolet light. Then, 5 µg Total RNA was reverse transcribed into cDNA according to the method of reverse transcription kit and SYBR Green real-time PCR amplification and detection was performed using an AB17500 fast system. The primer sequences of the target genes were as follows: forward, 5′-TGGGTTTCCCGTTGATGA-3′, and reverse, 3′-AGGGCTGCCTTCTCTTGT -5′, for GAPDH; forward, 5′-TGATCGGTTTCGTGAAGAGCT-3′, and reverse, 3′-ACGCACATTCCCAACACAAA-5′, for MRP2; forward, 5′-GTTTGGACTCAAGCACAGCA-3′, and reverse, 3′-TGAGTTTCCCAGAAGCCAGT-5′, for BCRP; forward, 5′-CTCACAGAGAGGGGTCGTTG-3′, and reverse, 3′-A

CTGTTAGCGGCAGTTTGGT-5′, for Claudin-1; forward, 5′-GGGGTGATTCGGATCCTGTC-3′, and reverse, 3′-TCCTCCAAAGATGCCCGTTC-5′, for Occludin; forward, 5′-C

CCTTACCTTTCGCCTGAAAC-3′, and reverse, 3′-CCTTCGTCTCTGAGCATCGT-5′, for ZO-1. For real-time PCR, the cycling conditions were as follows: 95 °C for 10 min, 45 cycles of 95 °C for 5 s, and 60 °C for 10 s, followed by a melting curve analysis-based assay with conditions of 95 °C for 5 s and 72 °C for 15 s, with an increase in temperature to 95 °C for 5 s. Targeted relative mRNA levels were normalized against GAPDH mRNA levels; The method of 2^-△△Ct^ was adopted to calculate the relative expression of MRP2, BCRP, Claudin-1, Occludin, and ZO-1.

### 2.9 Effect of HQ extract on tight junction by method of confocal immunofluorescence

Ileum segment of rats was embedded with paraffin and was cut into 5 μm slices, dew-axed and restored at 96 °C water bath. It was sealed for 1 h at room temperature with 5% rabbit serum, and then first antibodies were added, 4 °C overnight, second antibodies with FITC-labeled sheep anti-rabbit was added and was incubated for 1 h (avoid light), washed 3 times with 0.01 mol/L PBS and 5 min for one time. It was sealed with anti-fade mounting medium and observed by a laser co-aggregation microscope. Furthermore, semi-quantitative analysis method was used to analyze, the immunofluorescence image using an image pro plus, and in the image the green fluorescence represents the positive expression marker, while the blue fluorescence shows the nucleus and the IPP6.0 image software was used for semi-quantitative analysis of immunofluorescence images (Kang et al., 2017). The calculation formula is as follows (Liu et al., 2019, Zhang et al., 2015):

(1) $$\text{AOD}=\frac{\text{intDen}}{\text{Area}}$$

Where IntDen is the integrated optical density (IOD) of the images, Area is the fluorescence region of the images, and AOD is the average optical density.

### 2.10 Data analysis

All the data in the article is presented as the mean ± standard deviation (SD) from at least three independent experiments. Significant differences were calculated using One-way ANOVA, and statistical differences were considered significant, if *P* < 0.05.

## 3 Results

### 3.1 Evaluation of spleen deficiency syndrome models

The normal group rats, with a dense, smooth and shiny coats and brown granular feces, were responsive, normal in activity and diet. The model group showed curled and bunched, hunched back, narrowed eyes, dry and sparse hair, perianal filth, and even rectocele, with less food intake and slower weight gain than the controls (Table 1). According to the results of *D*-xylose and amylase activity, the two indexes in the spleen deficiency group were significantly decreased than those of the controls (Table 1).

**Table 1:** Evaluation of rats in normal group and model group (mean ± SD).

Evaluation index	Normal group (*n* = 5)	Model group (*n* = 54)
Amount of weight gain (per rats per day)	1.32 ± 0.38	0.75 ± 0.24^##^
Food-taking (per rats per day)	33.372 ± 2.25	27.376 ± 1.16^##^
*D*-xylose absorption	0.855 ± 0.15	0.484 ± 0.12^##^
Amylase activity	3223.7 ± 148.9	1396.0 ± 446.4^##^

#*P* < 0.05, ^##^*P* < 0.01 *vs* normal group

### 3.2 Effect of HQ on intestinal absorption of six alkaloids of FZ

**Fig. 1:**
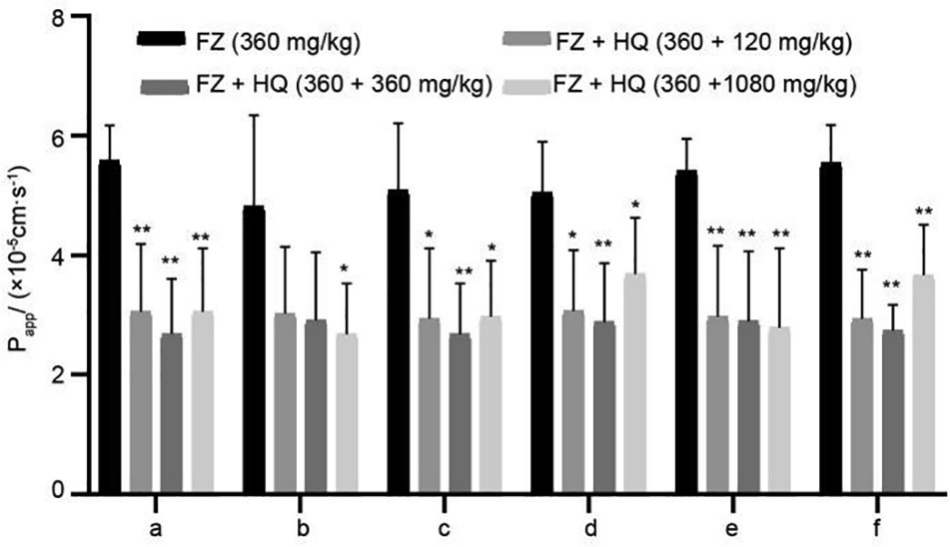
Effect of HQ on intestinal absorption of six ester alkaloids in FZ (mean ± SD, *n* = 5). a: benzoylhypaconie; b: benzoylmesaconine; c: benzoylaconitine; d: hypaconitine; e: mesaconine; f: aconitine. *P < 0.05, ^**^P < 0.01 *vs* model group.

**Fig. 2:**
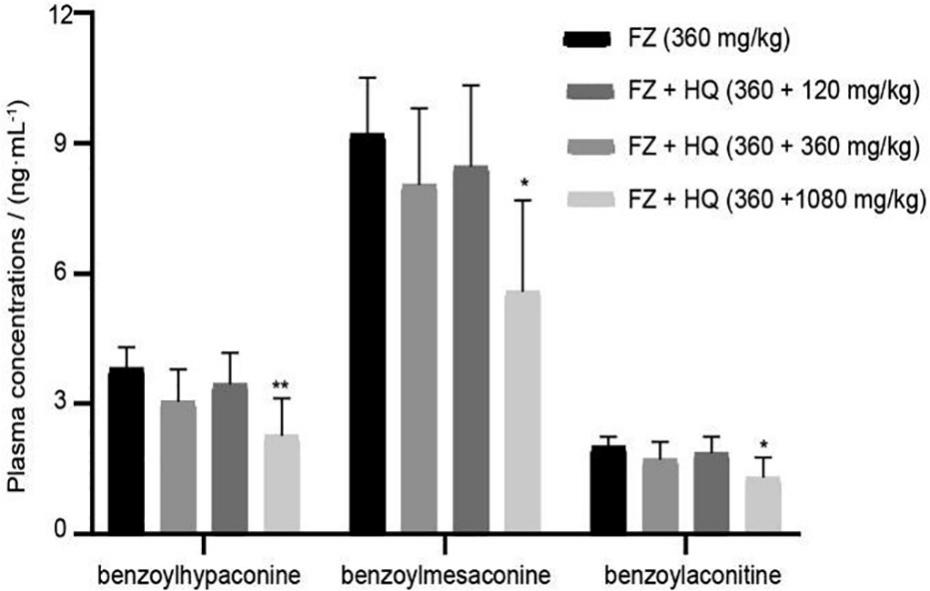
Effects of HQ on plasma concentrations of three monoester alkaloids in FZ in spleen deficiency rats (mean ± SD, *n* = 5). *P < 0.05, ^**^P < 0.01 *vs* model group.

The effect of HQ on the intestinal absorption of six alkaloids of FZ was shown in Fig. 1, and the effect of HQ on plasma concentrations of three monoester alkaloids in FZ in spleen deficiency rats were shown in Fig. 2. From Table 1, it was evident that different concentration of HQ can significantly reduce the intestinal absorption of six alkaloids of FZ, especially the medium concentration of HQ. The result of plasma concentrations verified that the high concentration of HQ could decrease the absorption of three monoester alkaloids compounds of FZ. Here only the plasma concentrations of three monoester alkaloids were detected because of the content of diester-type alkaloids in FZ was extremely low.

### 3.3 Effect of HQ on expression and mRNA levels of MRP2 and BCRP of spleen deficiency rats

**Fig. 3:**
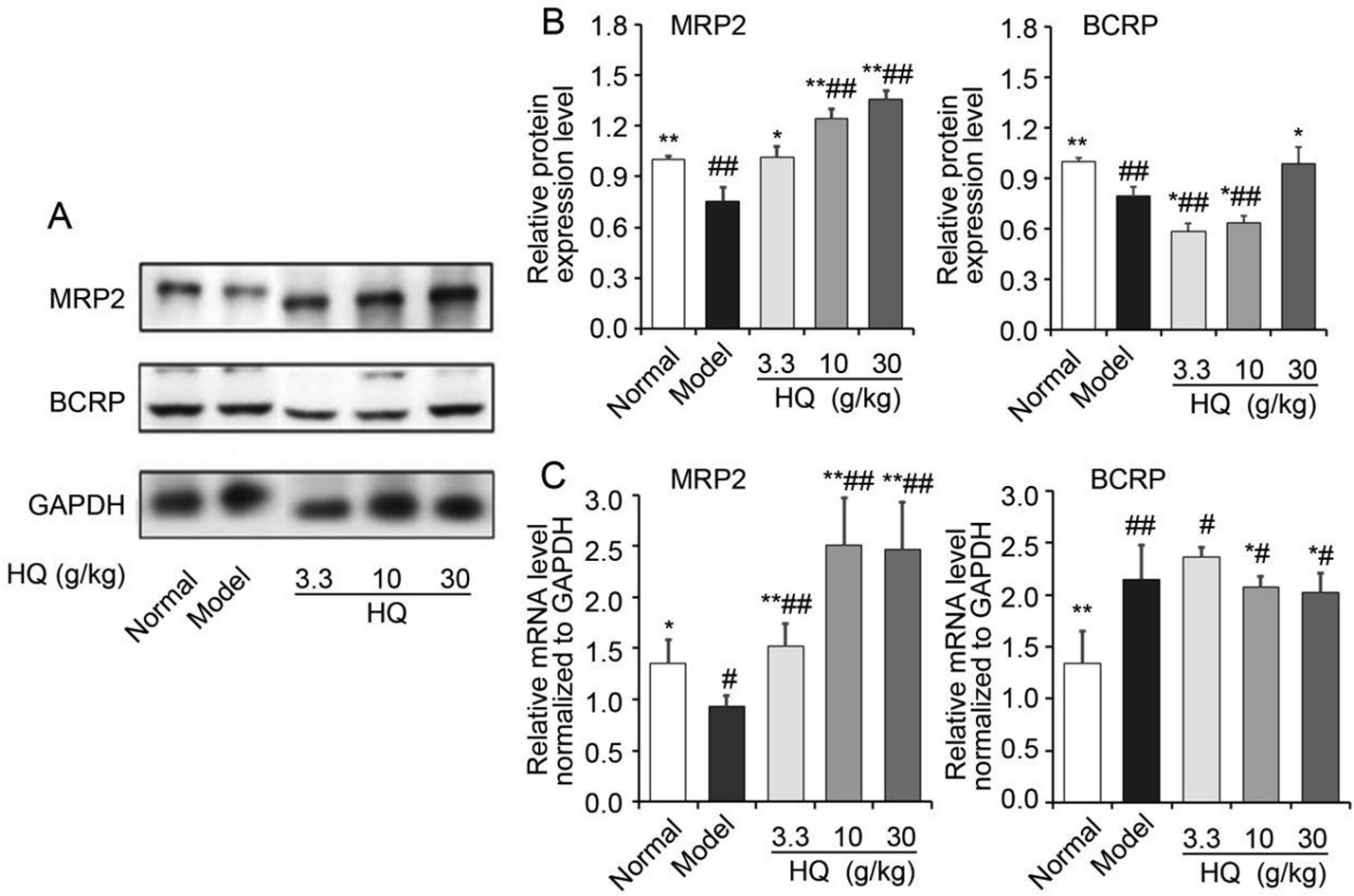
Effect of HQ on protein expression and mRNA levels of MRP2, and BCRP of spleen deficiency rats. (A) Protein levels of MRP2, and BCRP by Western blot analysis. (B) Aemi-quantitative results protein levels of MRP2 and BCRP by Western blot analysis. (C) mRNA levels of MRP2 and BCRP**.** *P < 0.05, ^**^P < 0.01 *vs* model group; ^#^*P* < 0.05, ^##^*P* < 0.01 *vs* normal group.

The effects of HQ on the protein levels of the studied ABC efflux transporters in the duodenal tissue of rats were shown in Fig. 3A and B. Compared with the normal group, the protein levels of MRP2 and BCRP in spleen deficiency models were significantly decreased (*P* < 0.05 or *P* < 0.01). While, compared with the spleen deficiency models (without oral administrations of HQ), the protein levels of MRP2 of models given different concentration of HQ were significantly increased (*P* < 0.05 or *P* < 0.01), and there was an obvious relationship between dosages. The protein levels of BCRP protein increased in the high-dose group of HQ. When given HQ, the functions of MRP2 and BCRP were increased after HQ administration; thus, the absorption of other compounds was decreased due to the increased function of the efflux proteins

The relative mRNA levels of MRP2 and BCRP were further analyzed in the duodenal tissue (Fig. 3C). Compared with the normal group, it indicated that the mRNA levels of MRP2 of the model group were significantly decreased (*P* < 0.05, *P* < 0.01), and the BRCP were also significantly increased. The results of mRNA levels of MRP2 were consistent with that of protein expression except that of BCRP.

### 3.4 Regulation of protein and mRNA levels of tight junction (Claudin-1, Occludin, ZO-1) by HQ

**Fig. 4:**
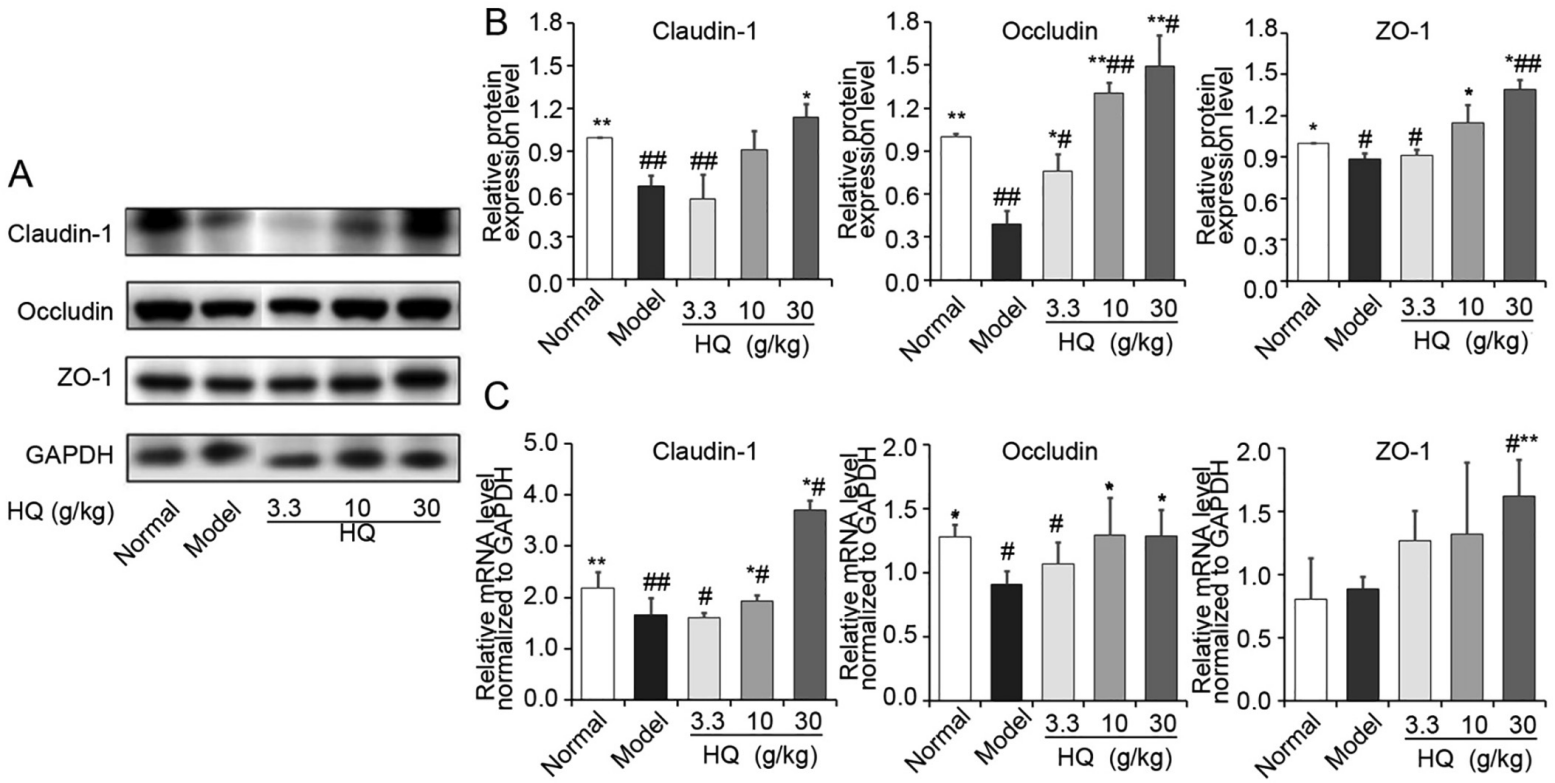
Effect of HQ on protein expression and mRNA levels of tight junction (Claudin, Occludin-1 and ZO-1). (A) Protein levels of Claudin-1, Occludin, and ZO-1 by Western blot analysis. (B) Semi-quantitative results of protein levels of Claudin-1, Occludin and ZO-1 by Western blot analysis. (C) mRNA levels of Claudin-1, Occludin and ZO-1. *P < 0.05, ^**^P < 0.01 *vs* model group; ^#^*P* < 0.05, ^##^*P* < 0.01 *vs* normal group.

As shown in Fig. 4A and B, compared with the normal group, the protein levels of Claudin-1, Occludin, and ZO-1 of spleen deficiency models were significantly decreased (*P* < 0.05 or *P* < 0.01) in model group. Compared with the model control group (without oral administration of HQ), the results showed that HQ could increase the protein levels of Claudin-1, Occludin and ZO-1 (*P* < 0.05 or *P* < 0.01). These tight junction proteins are involved in maintaining the permeability of the barrier next to the normal cell and so on, thereby prevent other foreign body including drugs from entering cells. When given HQ, the absorption of other compounds was decreased because HQ extracts enhanced the function of tight junction proteins.

The relative mRNA levels of Claudin-1, Occludin, and ZO-1 were further analyzed in the duodenal tissue (Fig. 4C). Compared with the normal group, the protein levels of Claudin-1, and, Occludin of spleen deficiency models were significantly decreased (*P* < 0.05 or *P* < 0.01) except that of ZO-1. Compared with the model group, it indicated that HQ could significantly (*P* < 0.05, *P* < 0.01) increase the mRNA levels of Claudin-1, Occludin, and ZO-1 in a dose-dependent manner.

### 3.5 Effect of HQ on expression and localization of three tight junction proteins by method of confocal immunofluorescence

**Fig. 5:**
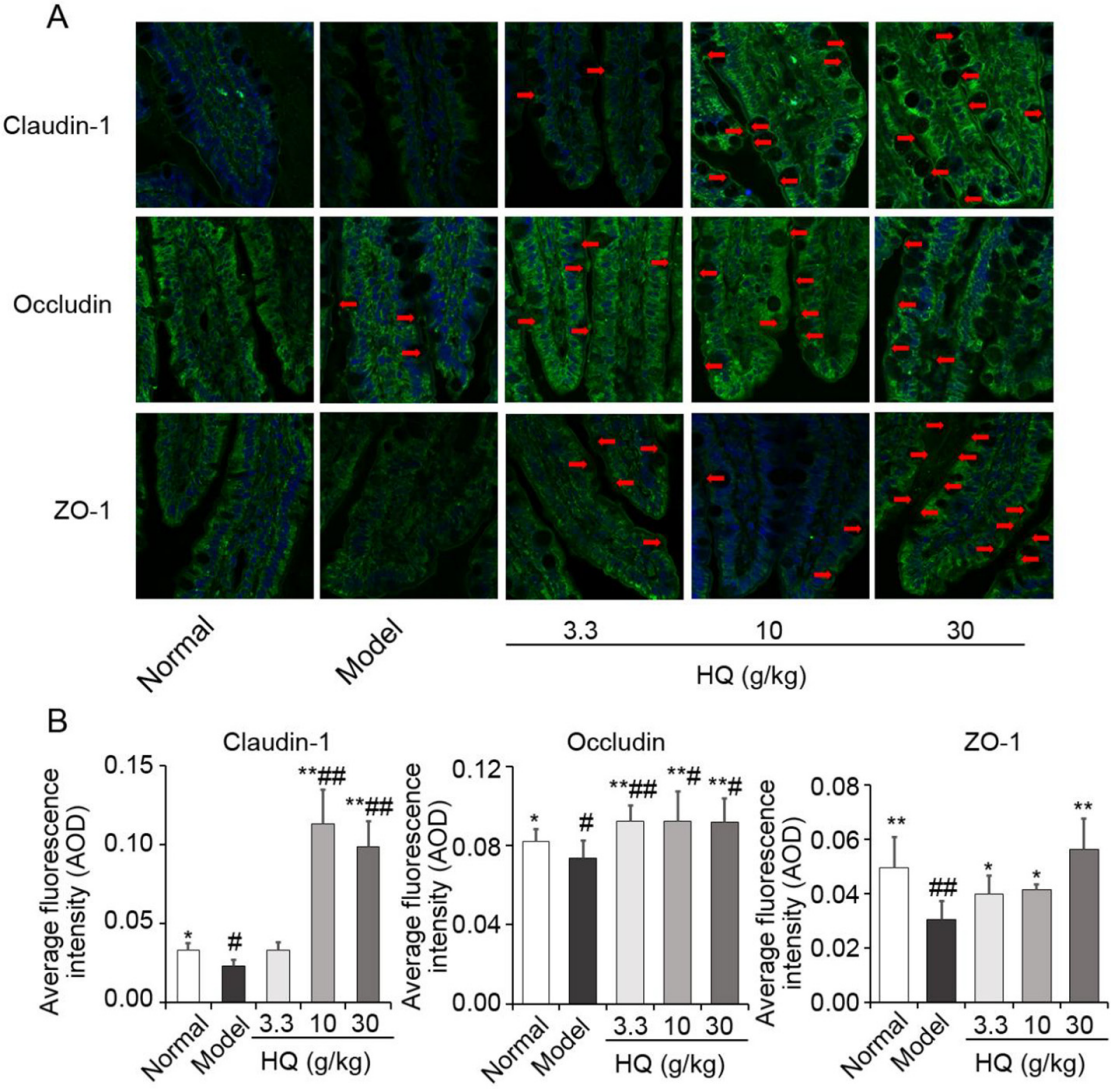
Localization of three tight junction (Claudin-1, Occludin and ZO-1) proteins. (A) Immunofluorescence images. (B) Average optical density of immunofluorescence. *P < 0.05, ^**^P < 0.01 *vs* model group; ^#^*P* < 0.05, ^##^*P* < 0.01 *vs* normal group.

In order to assess where the increased expression of tight junction proteins might be occurring in rats duodenal tissue after treatment with HQ, we fixed and stained the tissue with antibodies against Claudin-1, Occludin and ZO-1 and detected the expression of these proteins with a FITC secondary antibody using a confocal microscope, the tissue was imaged with a 40 × objective and DAPI was used to detect nuclear DNA of the cells. For the normal group, Claudin-1, Occludin and ZO-1 were typically located in the cytoplasm and membranes of monolayer columnar epithelial cells, while the cytoplasm of goblet cells was not stained with antibodies to these tight junction proteins (Fig. 5A). The 4–6 μm monolayer columnar epithelial cells are closely arranged and intercellular space could be seen faintly.

Compared with the normal group, the green fluorescence of Claudin-1, Occludin and ZO-1 of the model group enhanced in intestinal villi, and weakened in the goblet cells. The average optical density of immunofluorescence of Claudin-1, Occludin and ZO-1 was significantly decreased. After oral administration of HQ, the average optical density of immunofluorescence of Claudin-1, Occludin and ZO-1 was increased (Fig. 5B), these phenomena were most obvious in the medium and high dose group of HQ. So it could be concluded that the medium and high dose of HQ might effectively induct the expression of Claudin-1, Occludin and ZO-1, this may be one of the reasons that HQ inhibit the intestinal absorption of other drugs. What is more interesting is that the mucus layer among epithelial surface obviously thickens, which may be the other reason that HQ inhibit the intestinal absorption of other drugs.

## 4 Discussion

Modern research indicates that common traditional Chinese medicines believed to nourish *qi* and invigorate the spleen such as *Ginseng Radix et Rhizoma*, *Atractylodis Macrocephalae Rhizoma* and *Glycyrhizae Preparata Radix* can inhibit the absorption of diester alkaloids and reduce the strong toxicity of aconite (You et al., 2015, Zhang et al., 2013). Research indicates that *Atractylodis Macrocephalae Rhizoma* can reduce the toxicity of FZ by inducing CYP3A to improve the metabolism of FZ. In addition, a decoction of Four Mild Drugs was found to inhibit the absorption of endotoxins (Ji, Wang, & Li, 2016). Inhibition of absorption of harmful substances is a function of the intestinal barrier, which provides mechanical, microbial, chemical and immune barriers to drug absorption. The mechanical barrier consists mainly of intestinal mucosal epithelial cells and tight junctions. The microbial barrier consists of the bacterial flora lining the small intestinal mucosa that is beneficial to the host. The chemical barrier mainly refers to mucosal secretion and bactericidal or bacteriostatic constituents. The immune barrier is mainly composed of immune cells and immunoprotective proteins in the mucosa. These barriers could inhibit the absorption of harmful substances through mechanical interception, reduced permeability, killing and immune clearance of foreign organisms etc. In recent years a number of studies have reported that traditional Chinese herbal medicines believed to nourish *qi* and invigorate the spleen have the positive function of helping repair the mucosal barrier structure (Ji et al., 2016), chemical barrier (Zhang et al., 2014a, Zhang et al., 2014b), and balancing the microbiome of splenectomized animals (Ji et al., 2016), with evidence that these medicines decrease the mucosal permeability of harmful substances. All of this research supports the concept that the “Spleen is the master and defender” and herbal medicines believed to improve splenic function can also impact the efficacy of intestinal barriers (Zhang et al., 2014a, Zhang et al., 2014b, Ji et al., 2016).

HQ has the reputation of “invigorating *qi*” and is commonly used to provide *qi* to the spleen and lung. A major finding of this study is that HQ could significantly inhibit the intestinal absorption of six alkaloids of FZ in spleen deficiency rats. We found that the expression of MRP2 and BCRP was inhibited in the spleen deficiency rat model group, and HQ could up-regulate the expression of MRP2 and BCRP (Fig. 3). ABC transporters commonly act as drug efflux transporters and in tumor tissues, the over expression and activity of ABC transporters increases tumor cell resistance to drugs (Ceballos et al., 2019) (Zhitomirsky, Farber, & Assaraf, 2018). A second major finding of this study is that the expression of the three kinds of tight junction proteins was inhibited in the model group, HQ can significantly change the expression of tight junctions. As we all know, tight junctions play an important role at the boundary of the apical and basolateral plasma membrane regions to prevent foreign bodies from passing through the intercellular intestinal membrane (Bachinger et al., 2019). Tight junctions are made up of transmembrane proteins (including claudin and occludin) and cytoplasmic proteins (such as ZO-1, ZO-2, and ZO-3) (Mario Ortega-Olvera et al., 2018). Claudin-1 is a small transmembrane protein responsible for regulating the tight junction structure and function, which is critical for epithelial barrier integrity (Wang, Wang, Wang, Wan, & Liu, 2012). Occludin is a four transmembrane-helix integral membrane constituent protein of tight junction-strands (Cupri et al., 2015) and ZO-1 is a key cytoplasmic protein assembled at tigh junctions that binds to claudin and occludin as well as to the actin cytoskeleton (Ding et al., 2017). Passive diffusion through tight intercellular junctions exists in the transport of most small molecules, and alkaloids such as FZ are no exception. HQ increases the levels of mRNA and protein expression of the tight junction proteins Claudin-1, occludin, and ZO-1 in the intestine of rats with spleen deficiency, and potentially becomes the main barrier to prevent the internalization of solute through the paracellular pathway (Bachinger et al., 2019).

We found that administration of HQ could induce goblet cells of spleen deficiency rats to synthesize and secrete tight junction structural proteins and a thick layer of mucus was increasingly deposited on the surface of epithelium of the intestinal cavity (Fig. 5). The intestinal surface is covered with a protective mucus layer composed of mucoproteins which form a gel network structure. The composition and thickness of this mucosal layer determines the permeability of the mucus barrier to solutes and its protective efficiency against pathogens (Li & Yu, 2017). Since the amount of mucus was observed to significantly increasing with HQ administration significantly, the permeability of this barrier would likely reduce and its efficacy in preventing solute absorption would increase. Thus, our finding implicating treatment with HQ is upregulating three physiological mechanisms that could affect drug absorption, ABC transporters, the tight junction between intestinal epithelial cells and the gut epithelial mucosa.

Combined, the results of this study suggest that HQ can improve the physiological changes of intestinal environment in rats with spleen deficiency, which may lead to a decrease in the absorption of drug-like molecules, and the absorption of six alkaloids tested in this study are all inhibited. Our findings provide evidence for a possible mechanism by which HQ protects against FZ toxicity when used in traditional Chinese medicine as a herbal pair, such as in the Qifu Decotion (Zhang et al., 2018). A broader implication of these findings is that the uptake of a biologically active molecule, such as a modern anti-cancer drug, may be inhibited when used in combination with HQ. However, further research needs to be done to identify whether this property of HQ is specific to compounds such as the alkaloids tested in this study or is relevant to traditional or modern drugs in general.

Many ancient physicians have proposed that the “spleen is the master of the defense”, meaning that the spleen plays an vital role in maintaining the body’s equilibrium and limiting negative interactions with the external environment. HQ, a traditional medicine that has long been linked to healthy splenic function, appears to have more general properties in other organs that defend the body against external insult such as the small intestine. However, how mightly these traditional beliefs in Chinese medicine impact on modern medicine? Yan Li of the Ming Dynasty pointed out that “the spleen communicates with the small intestine” in his book of *Introduction to Medicine*. Base on this theory, the results of this study support that traditional Chinese medicines that promote the activity of the spleen also modify the function of the small intestine. The small intestine is not only an important organ for digestion and absorption of nutrients but also the first barrier for the body to resist endogenous and exogenous harmful substances (Greenwood-Van Meerveld, Johnson, & Grundy, 2017). Thus, the spleen role as defender of the body in traditional Chinese medicine and our understanding of the protective function of the small intestine in modern medicine could be linked, with the activity of traditional Chinese medicines such as HQ acting as conduits to promote both functions.

## 5 Conclusion

In summary, our results provide insight into a molecular mechanism for the efficacy of HQ in decreasing the intestinal absorption of six alkaloids of FZ in rats with spleen deficiency, inhibiting absorption function through regulating MRP2 and BCRP and Tight junction proteins expression, which can be effective in the spleen deficiency. This study provide support for the potential role of HQ as an intestinal barrier regulator to reduce toxicity for clinical therapy, and further studies are needed for the detailed investigation of the HQ regulation mechanism.

## Ethics approval and consent to participate

All animal studies were performed according to approved protocols and the guidelines of the Institutional Animal Ethical Care Committee (NIPER) and were approved by the local ethics committee for animal experimentation. All efforts were made to minimize the number of animals used and their suffering.

## Authors' Contributions

Xulong Chen and Xinli Liang drafted the manuscript. Zhenggen Liao conceived and designed the experiment. Xiaoqiang Kong and Miaomiao Ji contributed to the revisions of the manuscript. Abid Naeem, Cheng Li, Hao Zheng and Mingxia Gao contributed to the draft of the fundamental theories of traditional Chinese medicine of the review.

## Declaration of Competing Interest

The authors declare that they have no known competing financial interests or personal relationships that could have appeared to influence the work reported in this paper.
